# The Role of Distribution Forms of Fe–Cr–C Cladding Layer in the Impact Abrasive Wear Performance of Hadfield Steel

**DOI:** 10.3390/ma13081818

**Published:** 2020-04-12

**Authors:** Zhang Pan, Xuanpu Dong, Huatang Cao, Qiwen Huang

**Affiliations:** 1 State Key Laboratory of Materials Processing and Die & Mould Technology, Huazhong University of Science and Technology, Wuhan 430074, China; panz2012phd@hust.edu.cn (Z.P.); hmst66@163.com (Q.H.); 2 Department of Materials, University of Manchester, Manchester M13 9PL, UK

**Keywords:** Hadfield steel, PTA cladding, over-lapped cladding, dot-shaped cladding, Fe–Cr–C cladding layer, impact abrasive wear

## Abstract

To investigate the role of different distribution forms of Fe–Cr–C cladding layer in the impact abrasive wear performance of Hadfield steel, the over-lapped Fe–Cr–C cladding layer and dot-shaped Fe–Cr–C cladding layer were deposited, respectively, by plasma transferred arc (PTA) cladding on Hadfield steel. The microstructure, microhardness and impact abrasive wear performance of the two cladding layers under the impact of glass sand, granite and quartz sand were investigated. The results showed that both microstructures of the cladding layers were hypoeutectic Fe–Cr–C microstructures. The average microhardness of the over-lapped cladding layer and dot-shaped cladding layer was around 560 HV_0.2_ and 750 HV_0.2_, respectively. The over-lapped Fe–Cr–C cladding layer could only improve the impact abrasive wear resistance of the Hadfield steel under the wear condition of the glass sand. Meanwhile, the dot-shaped Fe–Cr–C cladding layer could improve the impact abrasive wear resistance of the Hadfield steel under all the three kinds of the abrasives because of the overall strengthening effect of its convex shape and the hypoeutectic FeCrC microstructure.

## 1. Introduction

Hadfield steels have been widely used in the field of the impact abrasive wear to produce wear-resistant components such as the jaw crusher plates and cone crusher liners because of their excellent toughness and impact hardening property [1,2,3]. The characteristics of the abrasive [4,5,6,7] such as hardness and compressive strength, have a significant influence on the impact hardening property of the Hadfield steel. When in contact with abrasives with a higher hardness and compressive strength, Hadfield steel will present a stronger impact hardening property but still at a sacrifice of more severe wear, leading to shorter service life. Therefore, it is crucial to improve the wear resistance of Hadfield steel.

When choosing the wear-resistant strengthening technologies for Hadfield steels, the characteristics of the abrasives should also be considered. The high hardness and compressive strength of the abrasive usually require the reinforcing layer to be strong and tough enough to survive [8,9]. The over-lapped strengthening technologies such as bimetal casting technology [10] are more suitable for wear applications with a soft abrasive counterpart because of the high strength but low toughness of the strengthening layer. However, discrete strengthening technologies such as cast-in technology [11] and discrete hardfacing technology [12,13] tend to meet the wear applications with both soft and hard abrasives because the strengthened layers possess combined superior strength and toughness.

Compared to cast-in technology, the cladding layers obtained by the discrete hardfacing technology are metallurgically bonded with the substrate to foster good adhesions. Thus, the cladding layers are not easy to peel off from the substrate. In addition, the discrete hardfacing technology is not affected by the casting process of the components, and it is also easy to design the suitable distribution form of the hardfacing layers on the surface of the components to match actual service conditions [12].

Due to its low thermal conductivity and high thermal expansion coefficient, Hadfield steel is sensitive to heat input, and carbides tend to precipitate in its heat-affected zone during the welding and hardfacing process [14,15,16,17]. The application of the discrete hardfacing technology for Hadfield steel could effectively control heat input in the hardfacing process to reduce the precipitation tendency of carbides from the austenite phases in the heat-affected zone. Besides, a heat source with a high energy density from hardfacing technologies such as PTA cladding technology [18] is also beneficial to reducing the heat input in the hardfacing process. Isakaev [17] used PTA cladding technology to deposit the over-lapped cladding layer on the Hadfield steel and found that there were no obvious carbides that precipitated in the heat-affected zone.

High chromium cast iron material systems are a common candidate for improving the wear resistance of components used in the field of the abrasive wear [10,11,19]. For hardfacing technology, the hypereutectic Fe–Cr–C hardfacing layer [19,20] is often used in the fields of high-stress abrasive wear and low-stress impact abrasive wear because of its high hardness and low toughness. In contrast, the hypoeutectic Fe–Cr–C hardfacing layer with a relatively higher toughness shows good application potential in the field of high-stress impact abrasive wear. The over-lapped hypoeutectic Fe–Cr–C hardfacing layer [21] deposited on the low carbon steel jaw plate showed superior wear resistance to that of the Hadfield steel jaw plat matrix when the hardness of the crushing material was about 500–600 HV_0.2_.

At present, there is no literature reporting on the difference between the application of the discrete hardfacing technology and the over-lapped hardfacing technology, to enhance the impact abrasive wear performance of Hadfield steel. Therefore, in this study, the over-lapped Fe–Cr–C cladding layer and the dot-shaped Fe–Cr–C cladding layer were deposited, respectively, by PTA cladding technology on the surface of the Hadfield steel. The microstructure, microhardness, and impact abrasive wear performance of the two cladding layers under the impact of glass sand, granite, and quartz sand were investigated.

## 2. Materials and Methods

### 2.1. Experiment Materials

**Figure 1:**
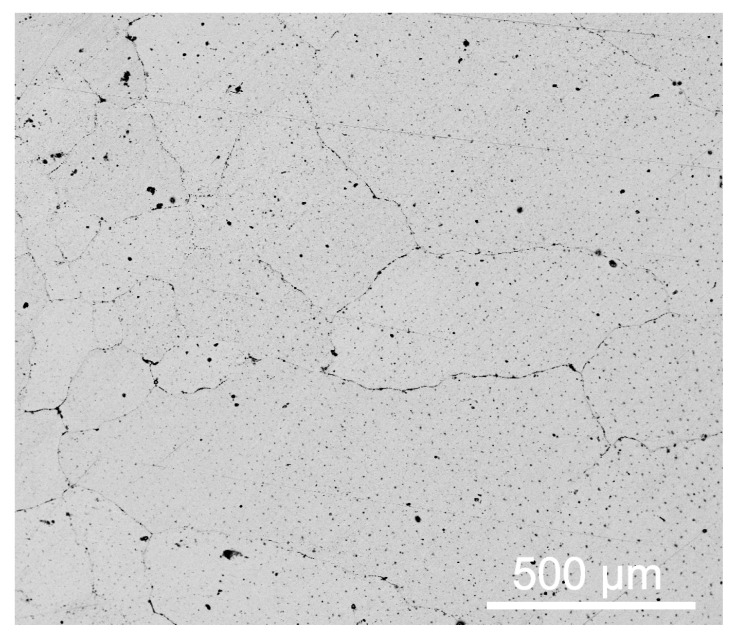
Scanning electron microscope (SEM) image of the microstructure of the as-received Hadfield steel.

Table 1 shows the main chemical composition of the as-received Hadfield steel substrate and the Fe-based cored wire for depositing the Fe–Cr–C cladding layer. The dimensions of the Hadfield steel substrate were 100 mm × 100 mm × 30 mm, and the diameter of the Fe-based wire was 1.6 mm. Before the PTA cladding treatment, the surface was polished thoroughly using 400-grit abrasive paper, and the surface was cleaned with alcohol to remove dirt. The microstructure of the Hadfield steel typically consists of a single austenite phase, as shown in Figure 1. The grain size of the austenite grains varied from 150 μm to 400 μm. The average measured surface hardness of the untreated substrate was approximately 180 HV_0.2_ to 220 HV_0.2._

### 2.2. Experimental Procedures

**Figure 2:**
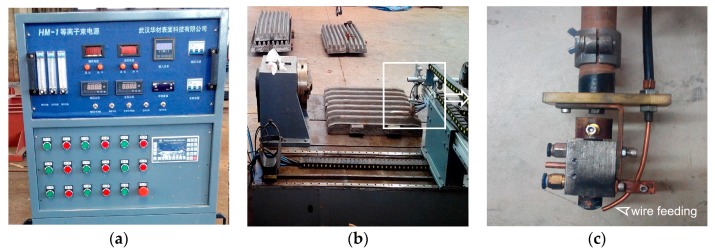
PTA cladding equipment: (**a**) Plasma power source and CNC controller; (**b**) Worktable; (**c**) Plasma torch.

PTA cladding equipment is shown in Figure 2. The main parts of the equipment are the plasma power source, CNC controller, plasma torch, worktable, wire feeding system, and the working gas system, respectively. The plasma arc consists of both the auxiliary arc (non-transferred arc) and main arc (transferred arc). Among them, the latter was used as the working arc in the experiment. The CNC controller could automatically control the on/off of the main arc. The cladding material was fed into the plasma arc by the wire feeding system, which was independent of the plasma torch, as shown in Figure 2c.

**Figure 3:**
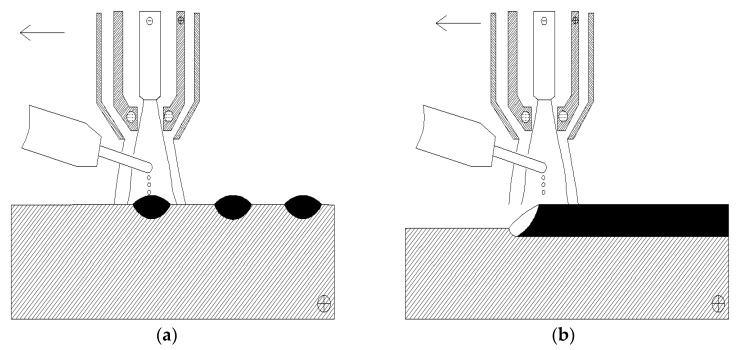
PTA cladding process: (**a**) The dot-shaped cladding (discrete dots); (**b**) The over-lapped cladding (continuous deposition).

The dot-shaped cladding and over-lapped cladding process by PTA cladding technology are shown in Figure 3a,b, respectively. The feature of the dot-shaped cladding process is that the plasma torch remains stationary during the wire feeding process (only moving to another place after one discrete dot cladding is finished). In contrast, the over-lapped cladding process is the conventional multi-track overlapping process (keeps moving for continuous deposition). The Fe-based wire remained electrically neutral in both of the two cladding processes.

It should be pointed out that optimized PTA cladding parameters are needed to guarantee that the microstructure of the deposited Fe–Cr–C cladding layer is the hypoeutectic Fe–Cr–C microstructure. In this experiment, the dot-shaped cladding layers on the surface of the Hadfield steel substrate were a rectangular distribution and the distance between the two adjacent dot-shaped cladding layers in the same direction was 10 mm. The diameter of the plasma torch nozzle was 3 mm, both the ionic gas and the shielding gas were argon, and the auxiliary arc current was fixed at 30 A. Other main optimized parameters are listed in Table 2.

After the PTA cladding treatment, transverse cross-sections were obtained from the processed samples by electric spark cutting. Specimens were mounted, grounded, polished, and then etched by 4 wt.% nital, sequentially. The microstructure was characterized using a Quanta 200 scanning electron microscope (SEM). The phases presented in the cladding layer were identified by an X’pert PRO X-ray diffraction (XRD) using CuKα generated at 40 kV and 40 mA under a scanning rate of 0.233°/s. Microhardness measurements across the top surface of the cladding layers towards the substrate were carried out using a TMVS-1 microhardness tester with a load of 1.96 N and a dwell time of 15 s for each.

**Figure 4:**
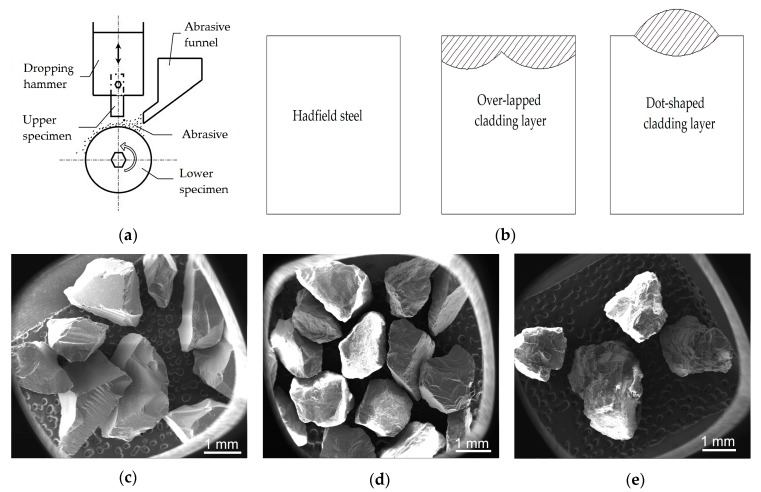
Impact abrasive wear test: (**a**) The schematic of the MLD-10 impact abrasive wear tester; (**b**) The schematics of the upper specimens; SEM images of (**c**) The glass sand; (**d**) The granite; (**e**) The quartz sand.

Wear tests were conducted using an MLD-10 abrasive wear tester, as shown in Figure 4. Impact energy of 1 J (corresponding to an impact hammer falling from a height of 10 mm) was used for the tests. The impact frequency was 150 times/min, and the overall impact time was 30 min. The lower specimen was GCr15 steel (macrohardness of 55 HRC) with a size of 15 mm (thickness) × Φ30 mm (inner diameter) × Φ50 mm (external diameter), Ra = 1.6 mm. The upper specimens were the tested material with a size of 10 mm × 10 mm × 30 mm, as shown in Figure 4b. The upper specimen of the over-lapped cladding layer consisted of two single cladding layers over-lapped with each other. At the same time, there was only one dot-shaped cladding layer on the surface of the upper specimen of the dot-shaped cladding layer. The three kinds of abrasive particles included glass sand (microhardness of ~500 HV_1_), granite (microhardness of ~800 HV_1_), and quartz sand (microhardness of ~1200 HV_1_), as shown in Figure 4c–e, respectively [9]. The average size of the abrasive was a 16–20 mesh, and the flowing rate was 40 kg/h. The wear loss was measured using a Mettler Toledo analytical balance with a weight accuracy of 0.1 mg.

## 3. Results

### 3.1. The Microstructure of the Cladding Layers

**Figure 5:**
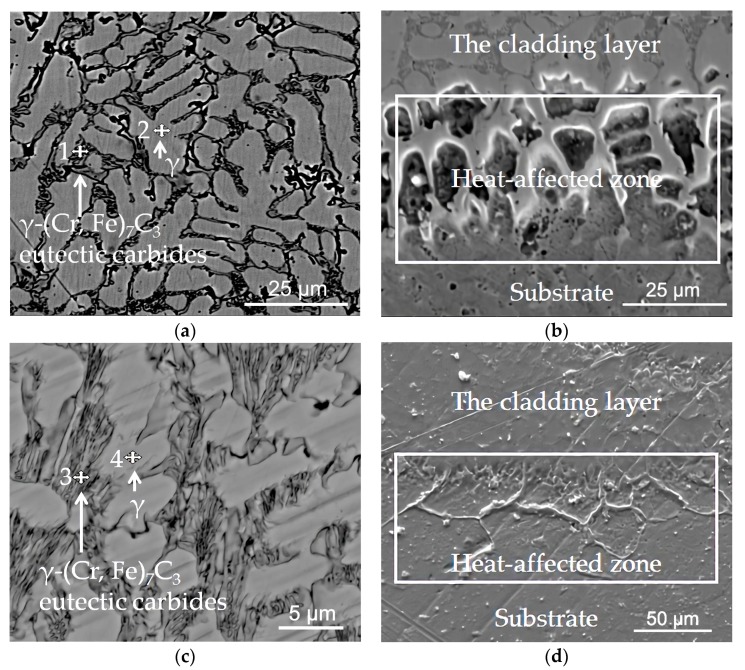
Microstructure of the cross-section of the two cladding layers: (**a**) The top zone of the over-lapped cladding layer; (**b**) The heat-affected zone and the substrate of the over-lapped cladding layer; (**c**) The top zone of the dot-shaped cladding layer; (**d**) The heat-affected zone and the substrate of the dot-shaped cladding layer.

The cross-section of the over-lapped cladding layer and the dot-shaped cladding layer both consist of three zones: the cladding layer, the heat-affected zone, and the substrate, as shown in Figure 5. The weld width, weld height, and penetration of the over-lapped cladding layer (for the single pass) and the dot-shaped cladding layer were approximately 6.5 mm, 1.0 mm, and 0.6 mm, and 5.0 mm, 1.2 mm and 0.4 mm, respectively. The dilution rate of the over-lapped cladding layer was slightly higher than that of the dot-shaped cladding layer. The heat-affected zone with a depth of about 30–100 μm was located between the bottom of the cladding layer and the Hadfield steel substrate. In this region, there was a phenomenon that the cladding layer grew into the Hadfield steel substrate along the grain boundary of the austenite of the substrate. This result was probably because the grain boundary of the austenite of the Hadfield steel at the bottom of the molten pool was melted into a liquid state due to its low melting point, and the molten liquid metal of the cladding layer flowed into the Hadfield steel substrate along the molten grain boundary of the austenite under the effect of the arc force and the thermal stress [17]. The cladding layer could combine firmly with the Hadfield steel substrate due to the existence of this heat-affected zone.

**Figure 6:**
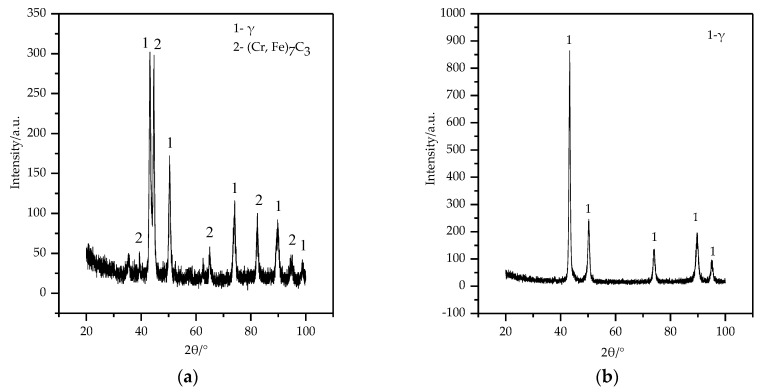
XRD patterns: (**a**) The over-lapped cladding layer; (**b**) The substrate.

Figure 5a,c shows that the microstructure of the two cladding layers consists of both a large number of dendrites and intercrystalline compounds except for some block or rod-like particles [19]. The EDS analysis of the dendrites and the intercrystalline compounds at the top zone of the two cladding layers are shown in Table 3. The contents of C, Cr, and Fe elements in the dendrites are slightly lower than in the intercrystalline compounds. Combined with the XRD analysis of the over-lapped cladding layer, as shown in Figure 6 below, it can be concluded that the microstructure of the two cladding layers consisted of a typical hypoeutectic Fe–Cr–C microstructure. Namely, the dendrites were the austenite, and the intercrystalline compounds were γ-(Cr, Fe)_7_C_3_ eutectic carbides.

Figure 5a,c also shows that the over-lapped cladding layer consists of coarser austenite and a lower number of the eutectic carbides than that of the dot-shaped cladding layer. This indicates that the dilution rate of the over-lapped cladding layer was higher than that of the dot-shaped cladding layer. In the multi-track overlapping process, the thermal input was higher, and the thermal accumulation was more evident than that of the dot-shaped cladding process. This resulted in a longer existing time of the molten pool, and thus, a longer growth time of the austenite and a higher dilution rate of the cladding layer.

### 3.2. XRD Analysis of the Cladding Layer

Figure 6 shows the XRD patterns of the over-lapped cladding layer and the Hadfield steel substrate. The XRD pattern of the dot-shaped cladding layer was not measured successfully because its convex shape rendered it difficult to collect the X-ray signal. The over-lapped cladding layer mainly composed of (Cr, Fe)_7_C_3_ and austenite (γ) phases, and the peak intensity of (Cr, Fe)_7_C_3_ phase was lower than that of the austenite phase. This indicated that the microstructure of the over-lapped cladding layer was the typical hypoeutectic Fe–Cr–C microstructure, while the predominant phase of the Hadfield steel substrate was the austenite.

### 3.3. Hardness Distribution

**Figure 7:**
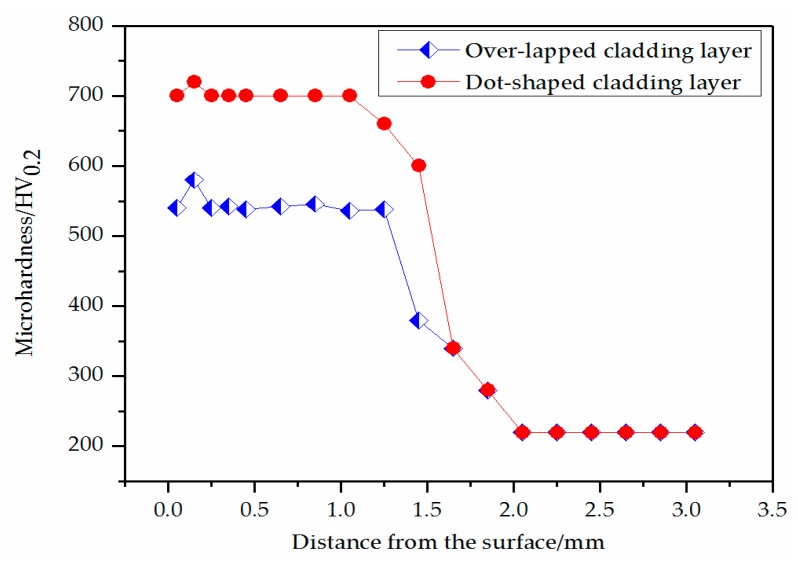
Hardness distribution of the two cladding layers along the depth direction.

The microhardness distributions of the two cladding layers on the surface of the Hadfield steel along the depth direction are shown in Figure 7. The average microhardness of the over-lapped cladding layer and the dot-shaped cladding layer was about 550 HV_0.2_ and 700 HV_0.2_, respectively. The presence of the oversaturated austenite phase and hard eutectic carbides in both cladding layers increased the hardness. Considering the lower content of the hard eutectic carbides and the coarser austenite grain, the microhardness of the over-lapped cladding layer was about 150 HV_0.2_ lower than that of the dot-shaped cladding layer.

### 3.4. Wear Performance

#### 3.4.1. Mass Loss

**Figure 8:**
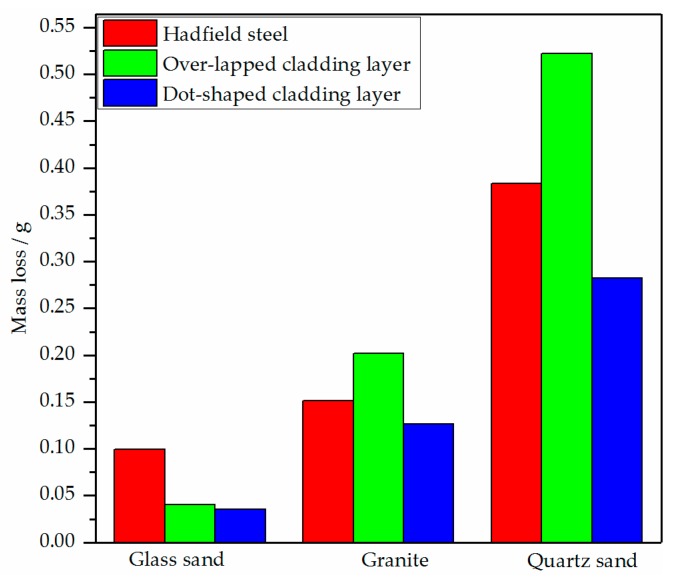
Mass loss of the specimens.

The mass loss of the two cladding layers on the surface of the Hadfield steel under the impact of the three kinds of abrasive particles is shown in Figure 8. With the increasing hardness of the abrasives, the mass loss of the two cladding layers and the Hadfield steel substrate increased. The dot-shaped Fe–Cr–C cladding layer on the surface of Hadfield steel reduced the mass loss under the impact of all three kinds of abrasive particles. Meanwhile, the mass loss of the over-lapped Fe–Cr–C cladding layer was only reduced under the impact of the glass sand.

#### 3.4.2. Morphologies of the Worn Surface

**Figure 9:**
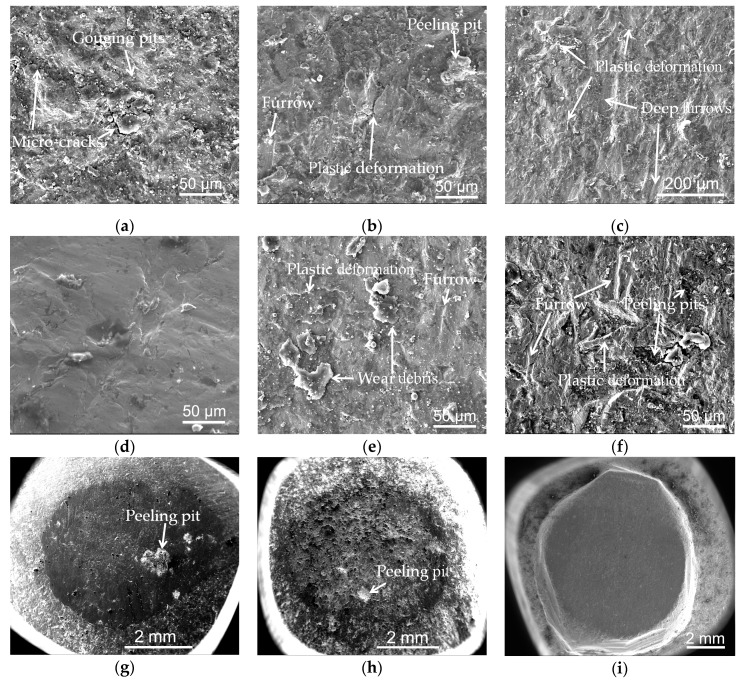

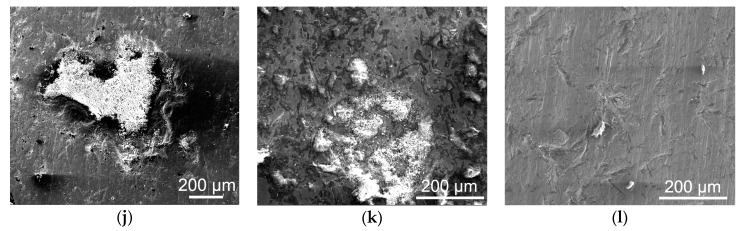
Morphologies of the worn surface: (**a**–**c**) The Hadfield steel: the glass sand, the granite, and the quartz sand, respectively; (**d**–**f**) The over-lapped cladding layer: the glass sand, the granite, and the quartz sand, respectively; (**g**–**i**) The dot-shaped layer: the glass sand, the granite, and the quartz sand, respectively; (**j**–**l**) Close-views of the top zone of the dot-shaped layer: the glass sand, the granite, and the quartz sand, respectively.

The morphologies of the worn surface of the Hadfield steel under the impact of the three kinds of abrasive are shown in Figure 9a–c. When the glass sand was used as the abrasive, there were a large number of shallow gouging pits and a few micro-cracks on its worn surface (see Figure 9a). In the case of the granite abrasive, there were plastic deformation ridges, furrows, and peeling pits on its worn surface (see Figure 9b). In terms of the quartz sand abrasive, the surface of the Hadfield steel suffered severe gouging and plowing effects by the abrasive, resulting in several short and deep furrows on its worn surface and also plastic deformation ridges (see Figure 9c).

The morphologies of the worn surface of the over-lapped cladding layer under the impact of the three kinds of abrasives are shown in Figure 9d–f. When the glass sand was used as the abrasive, there were only a few shallow furrows caused by the slight gouging and plowing of the abrasive on its worn surface (see Figure 9d).In the case of the granite abrasive, the surface of the over-lapped cladding layer suffered apparent plastic deformation, and there were wear debris and furrows on its worn surface (see Figure 9e). For the quartz sand abrasive, there were a large number of irregular peeling pits of variable size and depth on its worn surface. Moreover, short and deep furrows were distributed in the plastic deformation zone (see Figure 9f).

The morphologies of the worn surface of the dot-shaped cladding layer under the impact of the three kinds of abrasive are shown in Figure 9g–l. When the glass sand was used as the abrasive, the diameter of the worn surface on the top of the dot-shaped cladding layer was about 4.2 mm (see Figure 9g). The middle zone suffered a gouging effect by the glass sand, such that there was a large fatigue peeling pit. Meanwhile, the other areas on the top zone suffered a slight plowing effect caused by the glass sand (see Figure 9j). In the case of the granite abrasive, the diameter of the worn surface on the top of the dot-shaped cladding layer did not increase as compared to that of the worn surface caused by the glass sand (see Figure 9h). The whole worn surface on the top zone suffered the gouging effect caused by the granite, and there were enormous gouging pits and fine embedding granite particles on its worn surface (see Figure 9k). As for the quartz sand abrasive, the diameter of the worn surface on the top of the dot-like cladding layer increased to about 8.2 mm (see Figure 9i), and there were many long and shallow furrows on its worn surface (see Figure 9l).

#### 3.4.3. Morphologies of the Cross-Section of the Worn Specimens

**Figure 10:**
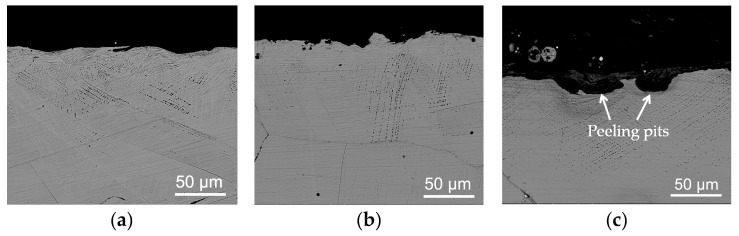

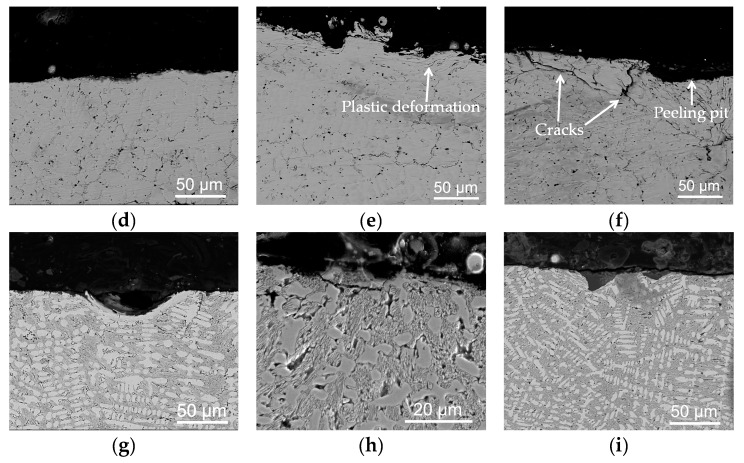
Morphologies of the cross-section of the worn specimens: (**a**–**c**) The Hadfield steel: the glass sand, the granite, and the quartz sand, respectively; (**d**–**f**) The over-lapped cladding layer: the glass sand, the granite, and the quartz sand, respectively; (**g**–**i**) The dot-shaped cladding layer: the glass sand, the granite, and the quartz sand, respectively.

The morphologies of the cross-section of the worn Hadfield steel specimens under the impact of the three kinds of abrasive particles are shown in Figure 10a–c. When the glass sand was used as the abrasive, the depth of the gouging pits was very shallow, and the surface was relatively flat (see Figure 10a). For the granite abrasive, the depth of the gouging pits increased, and the surface became uneven (see Figure 10b). For the quartz sand abrasive, there were relatively large peeling pits on its surface (see Figure 10c). With the increasing depth of the gouging pits, the density of the slip bands [5] in the austenite microstructure around the gouging pits became larger.

The morphologies of the cross-section of the worn over-lapped cladding layer under the impact of the three kinds of abrasive particles are shown in Figure 10d–f. When the glass sand was used as the abrasive, the top zone of the over-lapped layer was relatively flat, and no noticeable gouging pits were observed (see Figure 10d). In the case of the granite abrasive, the depth of the surface plastic deformation zone of the cladding layer was about 20–30 μm. There were a large number of peeling pits in this zone and the micro-cracks in the austenite microstructure under the peeling pits (see Figure 10e). For the quartz sand abrasive, the depth of the surface plastic deformation zone of the cladding layer increased to about 50 μm, and a few cracks were propagating the grain boundary of the austenite, as well as a large peeling pit in the top zone (see Figure 10f).

The morphologies of the cross-section of the worn dot-shaped cladding layer under the impact of the three kinds of abrasive are shown in Figure 10g–i. When the glass sand was used as the abrasive, the surface layer was relatively flat except for a small peeling pit with a diameter of around 0.08 mm (see Figure 10g). In the case of the granite abrasive, there were more small gouging pits on the top of the cladding layer and a few cracks in the subsurface layer (see Figure 10h). For the quartz sand abrasive, the surface layer was relatively flat except for a few gouging pits (see Figure 10i).

#### 3.4.4. Wear Mechanism

We conducted a comprehensive analysis of the morphologies of the worn surface, and the cross-section of the worn surface of the specimens. As such, the wear mechanism of the Hadfield steel substrate, the over-lapped cladding layer, and the dot-shaped cladding layer under the impact of the three kinds of abrasive particles are listed in Table 4.

The Hadfield steel had already suffered slight plastic deformation abrasion as the glass sand was used as the abrasive due to its low yield strength. However, the surface of the Hadfield steel had been hardly cut by the glass sand because its hardness increased to about 500–700 HV_0.2_ due to the impact hardening effect [5]. With the increase in hardness of the abrasive particles, the gouging and plough effects on the surface of Hadfield steel increased, and the degree of the plastic deformation abrasion and plough wear increased gradually.

The hardness of the over-lapped cladding layer was higher than the initial hardness of the Hadfield steel. Thus, the gouging abrasion resistance of the over-lapped cladding layer to the glass sand was more potent than that of the Hadfield steel, resulting in that the wear mechanism was slight gouging abrasion of the over-lapped cladding layer. This result follows that in the literature [21]. While under the impact of the granite with a 250 HV_0.2_ higher hardness than that of the over-lapped cladding layer, its wear mechanisms were plastic deformation abrasion and slight plough wear. The wear degree was more severe than that of the Hadfield steel. When the quartz sand was used as the abrasive, the plastic deformation abrasion and plough wear suffered by the over-lapped cladding layer was also more severe than those of the Hadfield steel because its overall combined strength and toughness was not as good as that of the Hadfield steel.

For the dot-shaped cladding layer, both of its wear mechanisms under the impact of the glass sand, and the granite were the gouging abrasion while the plough wear was the main wear mechanism when the quartz sand was used as the abrasive. There were two distinct differences between the dot-shaped cladding layer and the over-lapped cladding layer. One was that the microhardness of the dot-shaped cladding layer was about 150 HV_0.2_ higher than that of the over-lapped cladding layer, and the other was the convex shape of the dot-shaped cladding layer. The increase in the microhardness of the dot-shaped cladding layer was beneficial to improving its gouging resistance to the glass sand and granite, but not for the quartz sand. On the one hand, the convex shape of the dot-shaped cladding layer reduced its worn area, while on the other hand, the wear degree of the worn area became more severe due to the stress concentration in this area caused by the convex shape.

Both of the wear losses of the dot-shaped cladding layer under the wear conditions of the glass sand and the granite were lower than that of the over-lapped cladding layer. This was because the convex shape of the dot-shaped cladding layer allowed only its top area to be gouged by the abrasive, and its higher hardness increased the gouging resistance of the abrasive. When the quartz sand was used as the abrasive, the higher hardness and the stress concentration caused by the convex shape of the dot-shaped cladding layer would enhance the gouging and cutting effect on its worn area. Its wear loss still lower than that of the over-lapped cladding layer could be the results of its convex shape effectively reducing its worn area to decrease its wear loss.

## 4. Conclusions

The microstructure of the over-lapped cladding layer and dot-shaped cladding layer on the Hadfield steel substrate both consist of a hypoeutectic Fe–Cr–C microstructure. The average microhardness of the two cladding layers is 560 HV_0.2_ and 750 HV_0.2_, respectively.

The over-lapped Fe–Cr–C cladding layer could only improve the impact abrasive wear resistance of the Hadfield steel under the wear condition of the glass sand. Meanwhile, the dot-shaped Fe–Cr–C cladding layer could improve the impact abrasive wear resistance of the Hadfield steel under all the three kinds of the abrasive because of the comprehensive strengthening effect of its convex shape and the hypoeutectic FeCrC microstructure.

## Figures and Tables

**Table 1:** Chemical composition of the Hadfield steel and the Fe-based wire (wt.%).

Materials	C	Si	Mn	Cr	S	P	Fe
Hadfield steel	1.1	0.6	12.5	-	<0.05	<0.03	Bal.
Fe-based wire	3.5	1.2	1.5	22.5	-	-	Bal.

**Table 2:** Main parameters for the PTA cladding process.

Parameters	Dot-Shaped Cladding	Over-Lapped Cladding
Main arc, A	80	120
Torchworking distance, mm	8	8
Wire working distance, mm	2	0.5
The angle between the wire and the component, °	20	20
Wire feeding speed, mm·min^−1^	800	1200
Wire feeding time for per dot-shaped cladding layer, s	4	-
Plasma gas flow (Ar), L·min^−1^	2	2
Shielding gas flow (Ar), L·min^−1^	4	4
Plasma torch moving speed, mm·min^−1^	-	800
Over-lapped ratio, %	-	30

**Table 3:** EDS analysis of the four points in Figure 5 (at.%).

Points	C	Si	Mn	Cr	Fe
1	16.09	0.86	6.88	14.02	62.15
2	12.00	1.28	8.20	11.80	66.60
3	24.48	1.17	04.85	16.89	52.61
4	09.47	1.68	04.31	09.41	75.14

Note: the carbon content is indicative.

**Table 4:** Wear mechanisms.

Specimen	Glass Sand	Granite	Quartz Sand
Hadfield steel	Plastic deformation abrasion	Plastic deformation abrasion and plough wear	Plastic deformation abrasion and plough wear
Over-lapped cladding layer	Gouging abrasion	Plastic deformation abrasion and plough wear	Plastic deformation abrasion and plough wear
Dot-shaped cladding layer	Gouging abrasion	Gouging abrasion	Plough wear
